# Favorable Outcome With Eculizumab in Hemolytic Uremic Syndrome Presenting With Severe Neurological Complications: A Case Report

**DOI:** 10.1155/crin/8844271

**Published:** 2026-08-03

**Authors:** Maryam Zaitoun, Abdulkarim Alanazi, Tasneem Kattash, Abdullah Wali, Amna Abdelbagi, Sawsan Al Batati, Hassan Faqeehi, Saeed Al Zabali

**Affiliations:** ^1^ College of Medicine, Alfaisal University, Riyadh, Saudi Arabia, alfaisal.edu; ^2^ Pediatric Nephrology Department, Children’s Hospital, King Fahad Medical City, Riyadh, Saudi Arabia, kfmc.med.sa

**Keywords:** CNS involvement, complement-mediated TMA, hemolytic uremic syndrome (HUS), infection-associated HUS (STEC-HUS), seizures

## Abstract

**Introduction:**

Thrombotic microangiopathies (TMAs) are a group of rare, life‐threatening disorders characterized by a classic triad of MAHA, severe thrombocytopenia, and ischemic tissue injury. Thrombotic thrombocytopenic purpura (TTP) and hemolytic uremic syndrome (HUS) are the main types of TMAs. Based on the cause, HUS can be classified as typical or atypical.

**Case Presentation:**

We present the case of a 4‐year‐old child referred to King Fahad Medical City in Riyadh, Saudi Arabia, with complaints of bloody diarrhea, vomiting, and fever. The condition progressed to altered consciousness, seizures, and quadriparesis.

**Result:**

Upon admission, the patient received plasma infusion and underwent peritoneal dialysis. Eculizumab therapy was initiated 1 week after the presentation. Hematological and renal parameters improved rapidly, but neurological recovery was gradual, with significant progress observed over 6 years of follow‐up.

**Conclusion:**

This case highlights that severe neurological manifestations can be an initial feature of HUS, not just TTP. Eculizumab was effective and life‐saving in pediatric patients with TMA and severe neurological involvement, though CNS recovery may take years.

## 1. Introduction

Thrombotic microangiopathies (TMAs) are a group of rare, life‐threatening disorders characterized by a classic triad of microangiopathic hemolytic anemia (MAHA), severe thrombocytopenia, and ischemic tissue injury, most commonly acute kidney injury (AKI) [1]. Thrombotic thrombocytopenic purpura (TTP) and hemolytic uremic syndrome (HUS) are the main types of TMAs [2].

TTP is characterized by a pentad involving the triad of TMA, fever, and neurological symptoms [3]. TTP is caused by a gene mutation or acquired autoantibodies to ADAMTS13, a vWF metalloprotease that breaks down large vWF multimers into smaller ones [4, 5]. An activity level of less than 10% confirms the diagnosis of TTP in patients with evidence of hemolysis and thrombocytopenia [4].

HUS presents with the triad of thrombocytopenia, MAHA, and AKI [6]. The leading cause of HUS is Shiga toxin‐producing *Escherichia coli* (STEC), called typical HUS, contributing for 90%–95% of HUS cases [6]. Other infrequent causes, such as infections or genetic abnormalities that activate the alternative complement pathway uncontrollably, cause atypical HUS [6, 7].

Typical HUS usually occurs as a result of the consumption of contaminated food or drink and via person‐to‐person contact [7]. Of those infected with STEC, 5%–15% develop HUS, mainly affecting children below 5 years [7]. Ideally, they present with bloody diarrhea on day 2 or 3 after exposure, followed by HUS 3–10 days after the onset of diarrhea [7]. Vomiting (67%), fever (37%), and abdominal pain (29%) are other associated symptoms [8, 9].

In contrast to typical HUS, atypical HUS represents 5%–15% of HUS cases [10, 11]. Genetic mutations cause continuous activity of the alternative complement system at low levels [10, 11]. Inflammatory conditions, such as infections, cause endothelial damage and stimulate the activation of the coagulation cascade, resulting in TMA presentation [10, 11]. Complement‐mediated TMA can present with a spectrum of severity, from mild hematologic abnormalities to severe, life‐threatening end‐organ damage, such as gastrointestinal bleeding, seizures, blindness, or AKI requiring dialysis [10, 12, 13]. Unlike typical HUS, patients with atypical HUS often relapse after discontinuation of treatment, and kidney function does not recover without treatment [12].

Complement‐mediated TMA can present as idiopathic or secondary to a trigger, such as fever, upper respiratory tract infections (e.g., *S. pneumonia* [9], influenza virus [12], COVID‐19 [12]), HIV, autoimmune diseases, pregnancy [14, 15], non‐*E.coli* diarrheal illnesses, and drugs such as quinidine and chemotherapeutic agents [12, 16].

Although neurological manifestations are less common than renal manifestations, they are often associated with higher morbidity and mortality in HUS cases [17–19]. We aim to introduce the case of a patient in Saudi Arabia with HUS who had a good outcome despite severe brain involvement at the time of presentation.

## 2. Case Presentation

A 4‐year‐old girl was referred to King Fahad Medical City with a history of acute‐onset bloody diarrhea accompanied by vomiting and low‐grade fever. She subsequently developed seizures, altered consciousness, and quadriparesis. On admission, she was noted to have severe dehydration, oliguria, and a Glasgow Coma Scale (GCS) score of 7/15. Initial evaluation, including laboratory investigations (Table 1), blood morphology (Table 2), and urine examination (Table 3), revealed anemia, thrombocytopenia, and AKI. The patient was admitted to the pediatric intensive care unit (PICU) of a local hospital, where she received meticulous supportive care, including plasma infusion and peritoneal dialysis (PD). Upon arrival at our centre, she had severe neurological sequelae in the form of altered consciousness and quadriparesis. HUS and TTP are possible etiologies. Eculizumab therapy was initiated immediately, and a TMA workup was performed (Table 4).

**TABLE 1:** Laboratory investigation.

Test	Result	Normal range
White blood cells (WBC)	7.43	4.3–11.3 × 10^9^/L
Red blood cells (RBC)	3.03	4.3–5.3 × 10^12^/L
Hemoglobin	9.3 g/dL	11–15 g/dL
Mean corpuscular volume (MCV)	98.3	75–95 fL
Mean corpuscular hemoglobin (MCH)	30.7	24–30 pg
Hematocrit	29.8	35%–45%
Reticulocyte count	5.06	0.5%–1.5%
Platelet count	210	155–435 × 10^9^/L
Prothrombin time (PT)	13.3	9.7–12.6 s
Activated partial thromboplastin time (APTT)	19.1	25.3–38.3 s
INR	1.16	0.81–1.23
Serum creatinine	5.1 mg/dL	0.5–1.2 mg/dL
LDH	631	125–220 U/L

**TABLE 2:** Blood morphology.

Test	Results
Schistocytes	Slight
Anisocytosis	Slight
Hypochromasia	Slight
Poikilocytosis	Slight
Polychromasia	Slight
Tear drop cells	Slight

**TABLE 3:** Urine examination.

Test	Result	Normal range
RBC	66	< 5 cells/μL
WBC	104	< 10 cells/μL
Bacteria	Rare	—
Yeast	Absent	—
Microalbumin/creatinine ratio	5.8 g/mol	0–3.5 g/mol

**TABLE 4:** Immunology workup.

Test	Result	Normal range
C3 Complement	0.548 g/L	0.9–1.8 g/L
C4 complement	0.151	0.1–0.4 g/L
CH50	44 units	60–144 units
MPO	1.4	0–20
PR3	4.3	0–20
ADAMTS13 activity	13%	> 10 [SMA1]%
dsDNA	null	—

**FIGURE 1:**
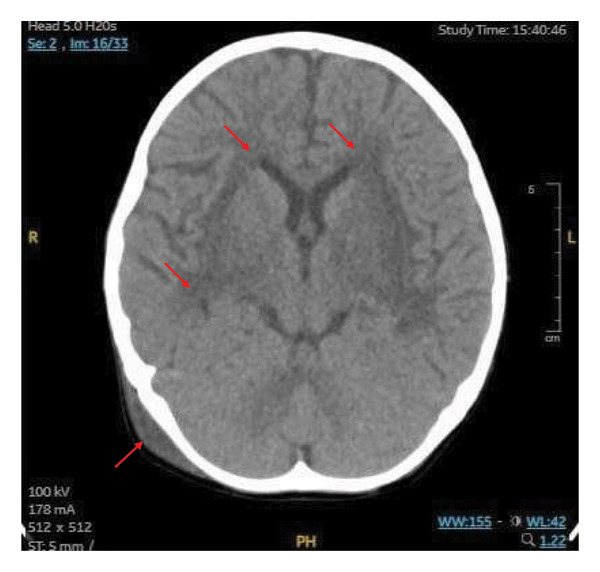
CT scan showing subcortical and periventricular hypodensities in the frontal and parietal lobe white matter involving the lentiform nucleus and internal capsule. There was a small subgaleal swelling over the right parietal bone.

**FIGURE 2:**
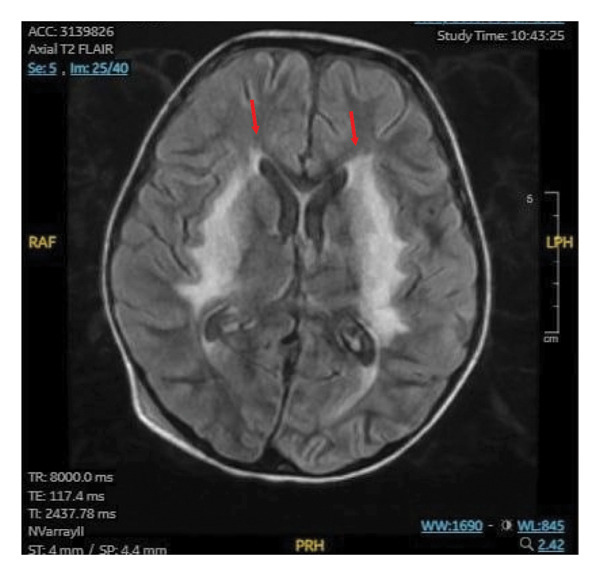
Confluent periventricular and subcortical FLAIR and T2WI hyperintense signal abnormalities were observed in the frontal and parietal lobe white matter. Furthermore, the external and extreme capsules, claustrum, and subinsular white matter bilaterally with few foci of cortical changes involving the right frontal lobe showed FLAIR and T2WI hyperintense signal changes.

**FIGURE 3:**
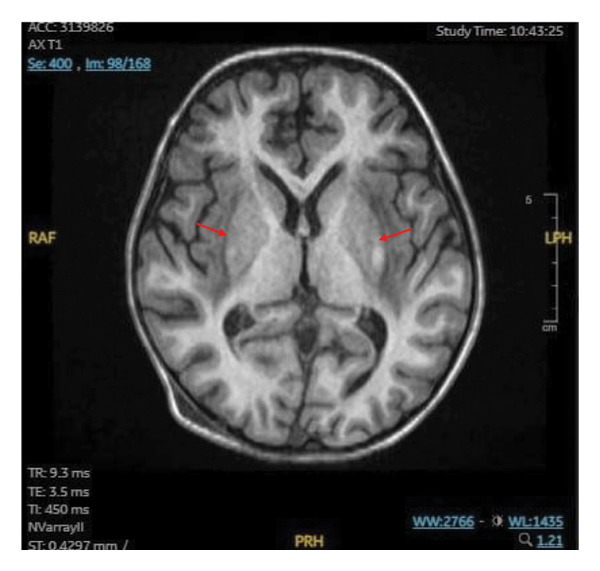
There are similar signal changes involving the outer putamen with an associated faint T1WI hyperintense signal abnormality.

Brain CT/MRI revealed diffuse hyperintense signal abnormalities in the periventricular white matter, basal ganglia, and cerebellum, consistent with CNS involvement in HUS (Figure 1), (Figure 2), (Figure 3). EEG performed during her ICU stay revealed slow background activity with diffuse paroxysmal discharges, prompting the initiation of anticonvulsant therapy.

Genetic testing, including the HUS panel, confirmed that she was heterozygous for deletions in the CFHR3 and CFHR1 genes. Eculizumab was administered for a total duration of 4 months. After receiving the genetic testing results and in accordance with the KFMC eculizumab discontinuation protocol, treatment was gradually tapered and discontinued.

### 2.1. Differential Diagnosis

Identifying the type of TMA can be challenging, but crucial to the treatment. This is due to other diseases that mimic its clinical presentation, most commonly TTP, STEC‐HUS, drug‐induced TMA, and disseminated intravascular coagulation (DIC) [20].

Given the patient’s age and unremarkable medication history, drug‐induced TMA can be excluded. In addition, the normal platelet counts, APTT, PT, and INR levels (Table 1) make DIC unlikely.

Since there are many forms of TMA, clinical manifestations alone will not be sufficient to establish the diagnosis of complement‐mediated TMA. Like all TMA subtypes, complement‐mediated TMA presents with the typical triad of thrombocytopenia, MAHA (manifesting as low hemoglobin, schistocytes, and elevated LDH levels), and AKI (high creatinine) [3, 20]. Therefore, further investigations that are particularly specific for each form of TMA should be performed.

In our case, ADAMTS13 activity was 13%, which is within the normal range, thereby ruling out a diagnosis of TTP. Although PCR for Shiga toxin is the diagnostic gold standard for STEC‐HUS, it was not available in our centre. The patient had a negative stool culture for E. coli, making STEC‐HUS less likely. In addition, serum complement C3 and C4 levels were measured. Complement C3 was significantly reduced, while complement C4 was in the lower side of the normal range (Table 4).

### 2.2. Treatment

The initial management for complement‐mediated TMA was supportive care [21]. Moreover, the management of complement‐mediated TMA may also include plasma exchange, eculizumab, and/or kidney‐hepatic transplantation [21]. Eculizumab is now considered the first‐line treatment for severe manifestations of complement‐mediated TMA [22].

Eculizumab, a humanized monoclonal antibody, binds to complement protein C5, inhibiting its cleavage [23]. This blocks the formation of the terminal complement components C5a and the membrane attack complex C5b‐9 [23]. Consequently, endothelial damage, thrombosis, and kidney injury are reduced in patients with complement‐mediated TMA [23]. Eculizumab should be started immediately, typically within the first 48 h of admission [24]. Our patient received a 600 mg IV infusion of eculizumab twice weekly.

### 2.3. Outcome and Follow‐Up

During her hospital course, she began to pass a good amount of urine, along with improvements in the hematological and renal parameters. PD was discontinued 2 weeks after initiation. Her neurological status gradually improved. Two months later, she had fully regained consciousness and demonstrated improved motor function; however, she remained quadriparetic and spastic. The patient continued eculizumab therapy at regular intervals in accordance with the HUS protocol.

She was subsequently admitted to a rehabilitation hospital, where she received extensive physiotherapy, occupational therapy, speech therapy, and swallowing management. After 3 years of follow‐up, she began to walk with support. Five years after her initial presentation, she remains clinically stable, with normal renal function and only mild neurological deficits.

## 3. Discussion

Differentiating between STEC‐HUS and complement‐mediated TMA is often challenging, especially in patients who present with bloody diarrhea and severe central nervous system involvement. Although the initial clinical features in this case were consistent with STEC‐HUS, gastrointestinal manifestations are not unique to STEC‐HUS and have also been reported in complement‐mediated TMA.

The lack of Shiga toxin polymerase chain reaction (PCR) testing is a limitation in this case. However, persistently low C3 levels, reduced total complement activity, and supportive, though nondiagnostic, genetic findings indicated complement dysregulation, which favored a complement‐mediated TMA. Although transient complement activation may occur in infection‐associated HUS, sustained hypocomplementemia is more typical of complement‐mediated TMA. The significant neurological and renal improvements observed after C5 inhibition further support the involvement of complement activation in disease pathogenesis. Collectively, these findings highlight the diagnostic overlap among HUS subtypes and emphasize the importance of integrating clinical course, complement studies, and treatment response in the management of severe TMA with central nervous system involvement.

Complement‐mediated TMA is caused by either a genetic mutation that causes uncontrolled stimulation of the alternative complement pathway or the production of autoantibodies [25, 26]. Neurological involvement is the most frequent extrarenal complication of complement‐mediated TMA [27]. In complement‐mediated TMA, the major pathological event is TMA, the main underlying cause of which is endothelial injury. TMA occurs systematically in the body; therefore, neurological involvement is a part of this systemic involvement [27].

This is a case report of a 4‐year‐old female who showed significant improvement despite presenting with severe signs and symptoms of HUS, including CNS involvement. Neurological manifestations in HUS cases are often associated with poor outcomes, such as long‐term physical disability, neuropsychological and cognitive sequelae, and even high mortality rates in up to 30% of cases [19].

In a single‐centre retrospective study, Kathrin Buder et al. reported 47 children with HUS who showed significant impairment in motor outcomes [28]. In 2023, a very similar case was reported in Egypt, which presented with bloody diarrhea, fever, dehydration, and decreased urine output, followed by generalized tonic‐clonic seizures, hypotonia, and episodes of unresponsiveness [17]. She had typical radiological findings and clinical manifestations of severe brain involvement [17]. The patient was started on phenobarbital with an immediate response, followed by further progressive improvement in her neurological condition after correcting her abnormal renal profile [17].

In a retrospective chart review conducted in 2021, it was established that 11% (22/202) of the enrolled STEC‐HUS patients had neurological manifestations, including encephalopathy, focal neurological deficit, and seizures. Only one patient died of multiple organ failure. Among survivors, 91% (19/21) regained full recovery. Most patients with CNS involvement with HUS who received eculizumab and/or plasma exchange fully recovered [19]. Similarly, Gulleroglu et al. revealed effective control and resolution of neurological manifestations in two patients with atypical HUS within 24 h of initiating eculizumab therapy [27].

Hushi Hu reported a 19‐month‐old child who presented with aHUS with severe neurological involvement, dilated cardiomyopathy, and renal impairment. Early eculizumab therapy resulted in significant improvements in the neurological state and normalization of cardiac and renal function [29].

Our case suggests that neurological involvement can be reversed with eculizumab, which is becoming the specific therapy for HUS, even in patients with HUS on dialysis.

Our patient was started on eculizumab, and 1 week later, urine output began to improve, along with renal function. In the condition of unavailability of ADAMTS13 testing, clinicians can use predictive scoring systems, such as the French or PLASMIC26 scores, to identify ADAMTS13 deficiency [30]. Both scores have a high negative predictive value (> 90%) for TTP [31]. The recent inclusion of the proteinuria‐to‐creatinuria ratio has further enhanced the diagnostic performance of these markers [32].

Identifying favorable outcomes in the disease would change the perception and expectations of physicians when they encounter these cases. Furthermore, it will open up more areas for research to discover common prognostic factors shared by these patients and attempt to apply them in clinical settings. Consequently, more accurate anticipation and counselling for the outcome of the disease could be identified.

## 4. Conclusion

Our patient experienced significant neurological and renal recovery following C5 inhibition with eculizumab, consistent with previously reported outcomes in HUS. Due to the absence of Shiga toxin PCR, it is difficult to differentiate between typical HUS and complement‐mediated HUS. This case illustrates the diagnostic challenges in differentiating HUS subtypes and supports the consideration of complement inhibition in selected cases with severe neurological involvement in HUS.

## Funding

No funding was received for this manuscript.

## Ethics Statement

The authors have nothing to report.

## Consent

The patient’s father allowed personal data processing, and informed consent was obtained from him.

## Conflicts of Interest

The authors declare no conflicts of interest.

## Data Availability

Data sharing is not applicable to this article as no datasets were generated or analyzed during the current study.
